# Van der Waals Photodetector with an Integrated WS_2_ Light-Harvesting Antenna

**DOI:** 10.1021/acsphotonics.5c00801

**Published:** 2025-09-17

**Authors:** Yesim Koyaz, Sotirios Papadopoulos, Antti J. Moilanen, Jonas D. Ziegler, Takashi Taniguchi, Kenji Watanabe, Lujun Wang, Lukas Novotny

**Affiliations:** † Photonics Laboratory, 27219 ETH Zurich, 8093 Zurich, Switzerland; ‡ Research Center for Materials Nanoarchitectonics, 31050 National Institute for Materials Science, 1-1 Namiki, Tsukuba 305-0044, Japan; § Research Center for Electronic and Optical Materials, 52747 National Institute for Materials Science, 1-1 Namiki, Tsukuba 305-0044, Japan

**Keywords:** two-dimensional materials, van der Waals photodetectors, transition metal dichalcogenides, graphene−WS_2_−MoSe_2_ heterostructures, responsivity enhancement, energy transfer, optical antennas, excitonic photodetection

## Abstract

The responsivity of graphene-based photodetectors can be improved by forming heterostructures with other 2D materials and by further coupling to nanoparticles or quantum dots. In this study, we demonstrate that the photoresponse of a Graphene/MoSe_2_/Graphene photodetector can be further enhanced by an external WS_2_ bilayer acting as a light-harvesting antenna. The WS_2_ bilayer is positioned outside the electronic pathway; thus, it does not directly contribute any photoexcited carriers. However, we observe a responsivity enhancement of up to 18 times, which can be explained by energy transfer from WS_2_ to graphene and the MoSe_2_ layer. Harnessing the excitonic properties of transition metal dichalcogenides (TMDs) as optical antennas defines a new strategy for photodetection.

## Graphical abstract

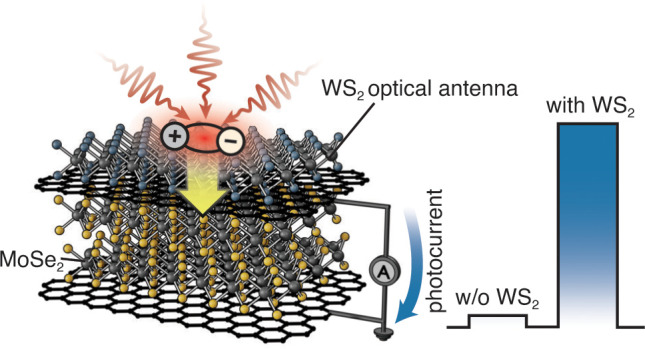

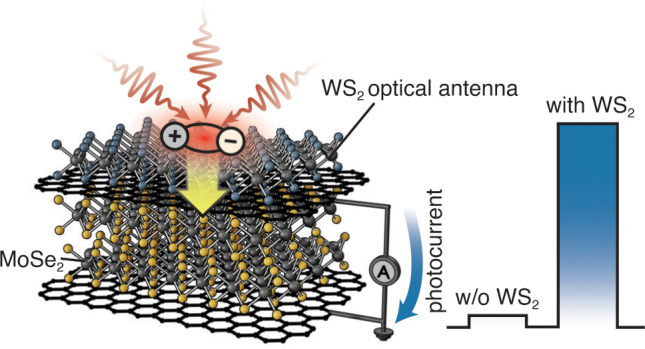

## I Introduction

In the last decades, 2D materials have attracted considerable attention for on-chip optoelectronics, including photodetectors, light-emitting devices (LEDs), and modulators. Graphene, in particular, stands out due to its unique band structure and ultrahigh mobility. However, its intrinsically low optical absorption (2.3%^1^) fundamentally limits the photodetection efficiency.^2^ Alternatively, Group-VI transition metal dichalcogenides (TMDs) exhibit stronger optical absorption especially with increased thickness.^3^ TMD-based photodetectors have demonstrated high responsivity, photogain, and detectivity.^4,5^ However, efficiently utilizing TMD photodetectors near the resonances remains challenging, as excitons must first dissociate and overcome their binding energy to contribute effectively to the photocurrent.

In this work, we introduce a so far unexplored strategy, namely using a TMD layer (WS_2_) as an optical antenna to enhance the local density of optical states (LDOS).^6,7^ This enhancement facilitates the generation of additional photocarriers in the photodetector, thereby improving its photoresponse. Recent studies have shown that light emission from van der Waals tunnel junctions can be significantly enhanced by incorporating a TMD layer on top of the device, leveraging excitonic interactions and a modified LDOS.^7,8^ Similarly, heterostructures such as monolayer MoSe_2_ coupled with monolayer WS_2_ and MoS_2_/hBN/WS_2_ heterojunctions within planar optical microcavities have demonstrated substantial photoluminescence enhancements due to increased LDOS, which promotes exciton recombination and emission.^9,10^

Building on these principles, we apply a similar approach to enhance the responsivity of a conventional Graphene/MoSe_2_/Graphene photodetector.^11–15^ Our results reveal a responsivity enhancement exceeding one order of magnitude, which we attribute to energy transfer from WS_2_ to the Graphene/MoSe_2_/Graphene heterostructure, effectively boosting the device’s photoresponse.

## II Device Structure and Fabrication

The cross-section of the fabricated van der Waals heterostructure is shown in Figure 1a. The device consists of MoSe_2_ sandwiched between two graphene layers, which serve as electrodes for carrier collection (Region A). The MoSe_2_ thickness influences both its absorption and excitonic properties, which consecutively determines the overall photoresponse of the heterostructure.^12,16^ A four-layer (4L) MoSe_2_ is chosen due to its indirect electronic bandgap, which helps mitigate radiative recombination of photogenerated carriers while maintaining short photocarrier transit times. Additionally, its thickness allows for the buildup of an electric field that facilitates efficient carrier collection while minimizing leakage currents.

**Figure 1:**
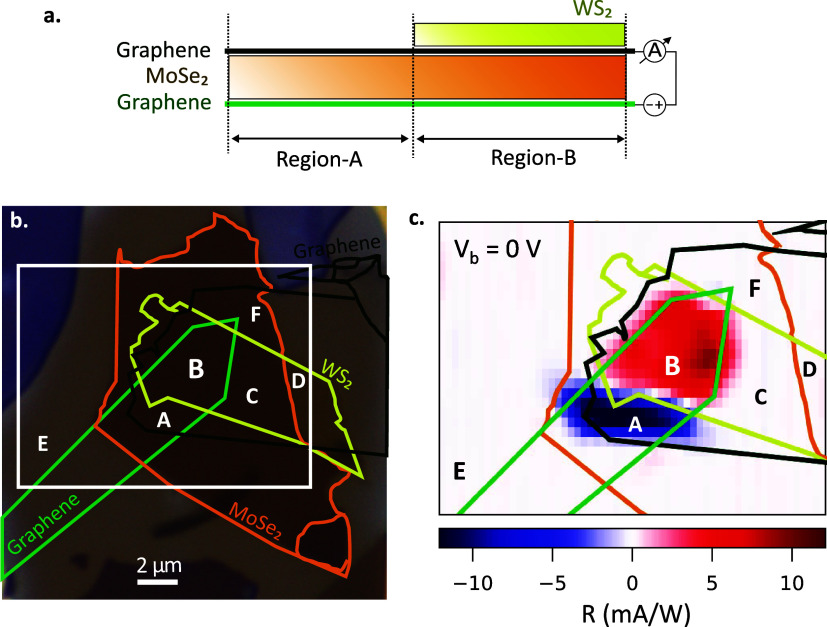
(a) Cross-sectional schematic of the van der Waals photodetector consisting of a Graphene/MoSe_2_/Graphene vertical junction without and with WS_2_ top layer (Region A and Region B respectively). (b) Optical image of the characterized photodetector consisting of 4L-MoSe_2_ and 2L-WS_2_. The outlines of the exfoliated 2D-flakes and the measurements’ scan area (white rectangle) are labeled. Region A and Region B are in accordance to (a). Region C, D, and F correspond to WS_2_/Graphene/MoSe_2_, WS_2_/Graphene, and MoSe_2_/Graphene heterostructures, respectively. Region E is outside of the heterostructure and labeled solely as a control region for reference. (c) Responsivity map at bias voltage *V*_b_ = 0 V and at illumination power *P*_in_ = 0.47 μW that has been recorded by raster-scanning the laser spot. The flakes’ outline and referred regions are in accordance to (a, b).

The graphene electrodes play a crucial role by reducing the potential barrier at the electrode-MoSe_2_ contacts as compared to conventional metal electrodes, ensuring efficient charge extraction.^17^ A bias voltage (*V*_b_) applied between the two graphene electrodes drives the separation of photogenerated electrons and holes, facilitating photoconductive operation.

In Region B, we introduce a bilayer (2L) WS_2_ on top of the heterostructure. We employ 2L-WS_2_ instead of a monolayer to reduce radiative recombination and increase the absorption of the overall system. The WS_2_ layer sits outside the photodetection pathway and, therefore, seems unlikely to contribute directly to photocarrier generation. However, it can modify the LDOS of the device, creating near-field channels through which its excitonic decay can optically couple to the Graphene/MoSe_2_/Graphene system, thereby influencing the photodetection process.

The following experimental analysis focuses on illumination with a HeNe laser at 633 nm near the A-exciton resonance of WS_2_ (∼630 nm). It is important to note that the WS_2_ layer is outside the electronic pathway, and there is no potential gradient applied across this layer.

The heterostructure is fabricated from mechanically exfoliated flakes using a dry pick-up and transfer process.^18^ The material stacking begins with the encapsulating hBN layer, followed by the transfer of graphene and MoSe_2_ layers. The MoSe_2_ thickness is determined to be four layers by optical contrast. Subsequently, the WS_2_ top layer is positioned such that it only partially covers the Graphene/MoSe_2_/Graphene heterostructure, enabling a direct comparison of the photoresponse with and without WS_2_. Finally, the completed device is transferred onto prepatterned Au electrodes and wire-bonded to copper pads for photocurrent measurements. A microscope image of the device is shown in Figure 1b. In the following section, we compare the photoresponse of Region A and Region B (without and with the WS_2_ top layer).

## III Experimental Results and Discussion

### III.I Performance with/without WS_2_ Antenna

To characterize the photoresponse, we illuminated the sample with a focused HeNe laser beam (633 nm), using a 0.8 NA 50× objective. The bias voltage (*V*_b_) is applied through a sourcemeter system (Keithley) and the current is simultaneously measured. The laser spot is raster-scanned by a piezostage. In Figure 1b, the scan area is marked with a white rectangle and the investigated heterostructure with and without WS_2_ are labeled as Region B and Region A, respectively, in accordance with the cross-section given in Figure 1a. To double-check the absence of any unintentional contributions on the photoresponse, the positions with WS_2_/Graphene/MoSe_2_ (Region C), WS_2_/Graphene (Region D), and MoSe_2_/Graphene (Region F) heterostructures are taken into consideration. Additionally, the region outside of the device (Region E) is also labeled to assess the electrical noise introduced by the electro-optic setup.

In Figure 1c, we present the responsivity (*R*) at *V*_b_ = 0 V over the scan area. We calculate *R* as the ratio of the measured photocurrent (*I*_ph_) over the illumination power at the sample position (*P*_in_). We observe that R is negligibly small in Region C, D, E, and F as well as in the flake edges, verifying that the active area of this device is solely composed of Regions A and B. The presence of photovoltaic photoresponse in Region A and B indicates that the two graphene electrodes (top and bottom) have slightly different doping levels. Moreover, the photoresponse has opposite polarity between Region A and B, which we attribute to additional doping due to charge redistribution at the contact between graphene and WS_2_.^17^ Consequently, we observe near-zero photoresponse near the WS_2_ flake edge, as it is the boundary for Regions A and B. We also acknowledge that the photoresponse of the active area has slightly leaked out from all edges (including but not limited to Regions C and F) possibly due to our diffraction-limited system as well as possible charge and exciton diffusion.

Next, photocurrent (*I*_ph_) maps for various *V*_b_ values are shown in Figure 2a (*V*_b_ = 0.3 V) and Figure 2b (*V*_b_ = −0.3 V). The photocurrent maps at lower *V*_b_ values are presented in Supporting Information Section I. In all cases, we observe a significantly higher *I*_ph_ in Region B compared to other regions. The high *I*_ph_ observed under both bias polarities confirms the photoconductive nature of the photoresponse. Additionally, we detected an exceptionally high *I*_ph_ at a distinct spot within Region B, which may result from a localized defect or inhomogeneity introduced during the stacking process. The *I*_ph_ in Region A is spatially uniform but exhibits a low signal-to-noise ratio. As a noise reference, we consider the values from Region E. A notable *I*_ph_ appears in the left edge of Region C, likely due to leakage from Region B, potentially caused by the diffraction-limited nature of the setup, edge-related stray fields, or exciton/charge diffusion. Additionally, we acknowledge the nonzero *I*_ph_ observed in Region F (Graphene/MoSe_2_ stack), which we attribute to stray fields originating from Region B and the noise-limited resolution of this measurement. We do not observe any significant *I*_ph_ in Region D (Graphene/WS_2_ stack), thereby ruling out the potential contribution of photocarrier accumulation in graphene due to the presence of WS_2_. Overall, these results confirm an enhanced photoresponse throughout Region B, with a comparative assessment of the performance across Regions A to F.

**Figure 2:**
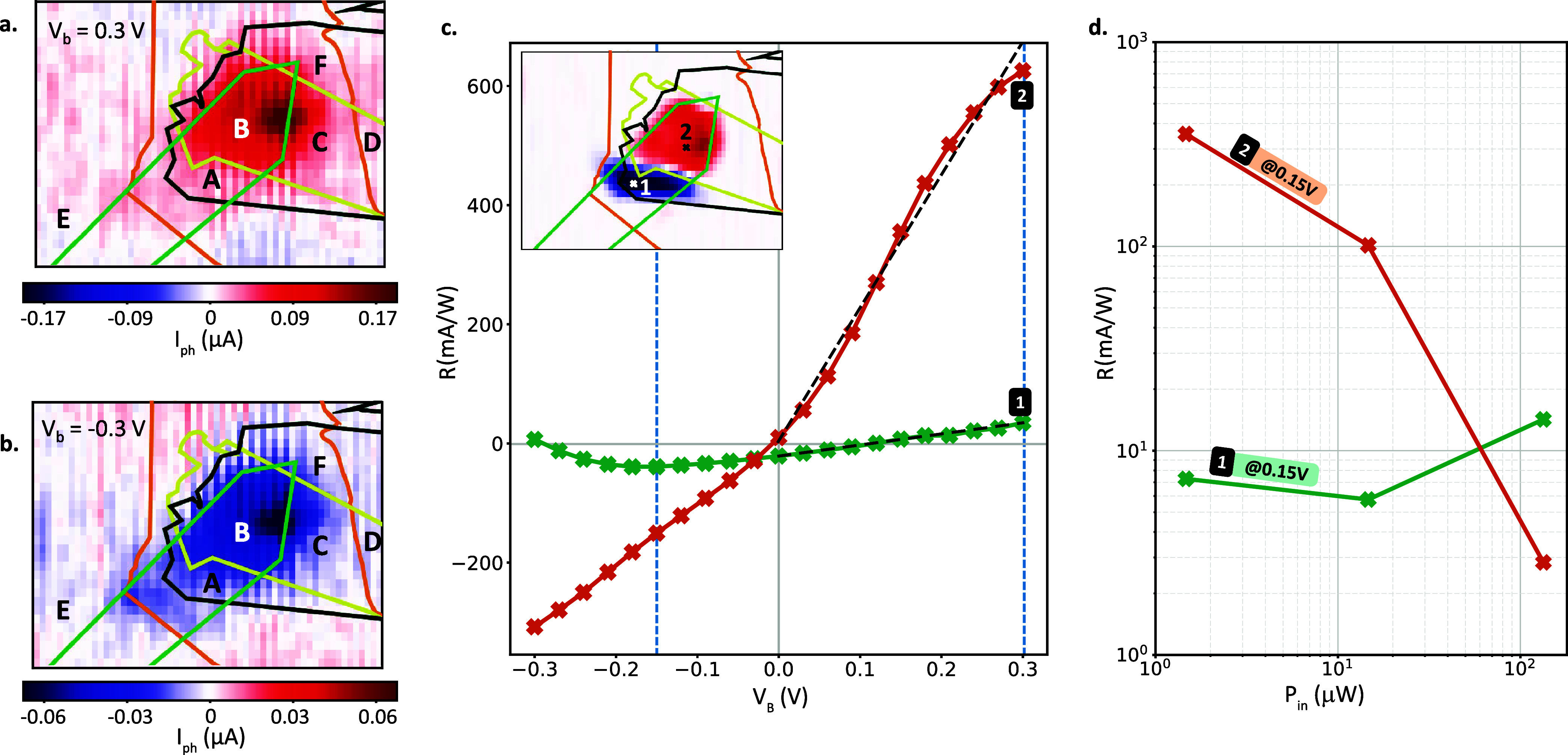
(a, b) Photocurrent maps for a bias voltage of *V*_b_ = 0.3 V (a) and *V*_b_ = −0.3 V (b) recorded by raster-scanning the laser spot. The flakes’ outlines and referred regions are in accordance to Figure 1b. (c) *R* as a function of *V*_b_ at locations with WS_2_ (Location-2 in Region B) and without WS_2_ (Location-1 in Region A) at *P*_in_ = 1.47 μW. The blue dashed lines are indicating the *V*_b_ values of maximum for both curves. These values are used to calculate the *R*-enhancement (i.e., the ratio of maximum *R* in Location-2 to Location-1). The black dashed lines are the linear fits of *R* for *V*_b_ > 0 at each location. Inset: Location-1 and Location-2 shown on top of the photocurrent map presented also in Figure 1c. (d) *R* at *V*_b_ = 0.15 V evaluated for different excitation powers of *P*_in_ for both locations.

To quantitatively compare the performance of the device with and without WS_2_, we measure the responsivity *R* at *P*_in_ = 1.47 μW as a function of *V*_b_ at selected locations, namely Location-1 and Location-2 as shown in Figure 2c. We observe the zero-responsivity point (*R* = 0) at *V*_b_ ≈ 0.12 V in Location‑1 and *V*_b_ ≈ −7 mV in Location-2. Consequently, *R* has an opposite polarity at *V*_b_ = 0 V, consistent with Figure 1c. Moreover, for *V*_b_ > −0.18 V, the positive slope (d*R*/d*V*_b_) in both locations is consistent with the photoconductive operation. However, for *V*_b_ < −0.18 V in Location-1, we observe a negative slope which we attribute to the high electrical noise becoming comparable to the photoresponse (as discussed for Figure 2a,b). Furthermore, we acknowledge the high electrical noise observed at high *V*_b_, especially affecting *R* in Location-1. We attribute this noise to possible leakage current from the ground terminal or crosstalk within the supply circuitry, measurement components, and piezostage. Even though the noise is present throughout the scan area, Region B exhibits a consistently stronger photoresponse.

We analyze the responsivity enhancement (*R*-enhancement), defined as the ratio of *R* in Location-2 to that in Location-1, at high *V*_b_. We find a value of ∼18 at *V*_b_ = 0.3 V and a value of ∼4.5 at *V*_b_ = −0.18 V (blue dashed lines in Figure 2c). For further comparison, we perform linear fits for *V*_b_ > 0 V (black dashed lines in Figure 2c) where the fit slope (d*R*/d*V*_b_) is 12× larger in Location-2. These results indicate that the introduction of WS_2_ leads to a significant enhancement in the responsivity under both bias polarities, which cannot be solely explained by differences in doping levels between the two regions.

In Figure 2d, we obtain *R* at different *P*_in_ at the investigated locations (namely Location-1 and Location-2) at *V*_b_ = 0.15 V. In Location-1, *R* remains mostly unaffected by increasing *P*_in_ (to an order of magnitude) whereas in Location-2, it exhibits ∼10-fold decrease per decade with increasing *P*_in_. This allows us to conclude that in the absence of WS_2_, the Graphene/MoSe_2_/Graphene system does not reach its saturation power regime in the investigated range. The absorption saturation of MoSe_2_ is prominent only at high optical power densities, where the conduction band becomes highly populated with photocarriers. Additionally, an increase in photocarrier density can enhance the built-in electric field across the heterostructure, resulting in dielectric screening that may limit the photoresponse at high power levels.^12,14,15^ However, there are no indications of these effects on Location-1. Therefore, the observed drop of *R* in Location-2 suggests the presence of additional free-carrier generation channels that are saturating the photoresponse already at low powers, indicating a trade-off between *R*-enhancement and high-power operation.

To further investigate the mechanism behind the *R*-enhancement, we characterize a second sample consisting solely of the WS_2_/Graphene/MoSe_2_/Graphene region. Microscope images and photocurrent maps of the sample are shown in the Supporting Information Section I. Additionally, in this device, we perform spectral responsivity measurements by varying the excitation wavelength using a broadband halogen lamp, with the emission filtered through a set of bandpass filters. Specifically, we use various filters with distinct central wavelengths and full-width-half-maximum (FWHM) values. By illuminating the sample through each filter and sweeping the excitation power, we extract the responsivity across a range of low-power excitation levels. The results are summarized in Figure 3a. A key observation is that similar to the previous device, the responsivity exhibits a strong dependence on excitation power, following a power-law behavior with an average slope of approximately −0.6 in the log–log scale. Furthermore, for wavelengths near the WS_2_ absorption region, we observe a substantial increase in responsivity, indicating that the enhancement originates from the presence of WS_2_. This behavior is clearly illustrated in Figure 3b, where we plot the responsivity as a function of filter wavelength for two representative input powers. The vertical bars indicate the corresponding FWHM values of each filter. Notably, the responsivity shows a drop beyond the A-exciton resonance of WS_2_ (630 nm), while there is a less pronounced and less conclusive indication of reduced response at shorter wavelengths, potentially suggesting a resonant behavior. These spectral response measurements reinforce the interpretation that WS_2_ enhances the photoresponse by acting as an optical antenna that selectively couples its excitonic energy to the active photodetection layers.

**Figure 3:**
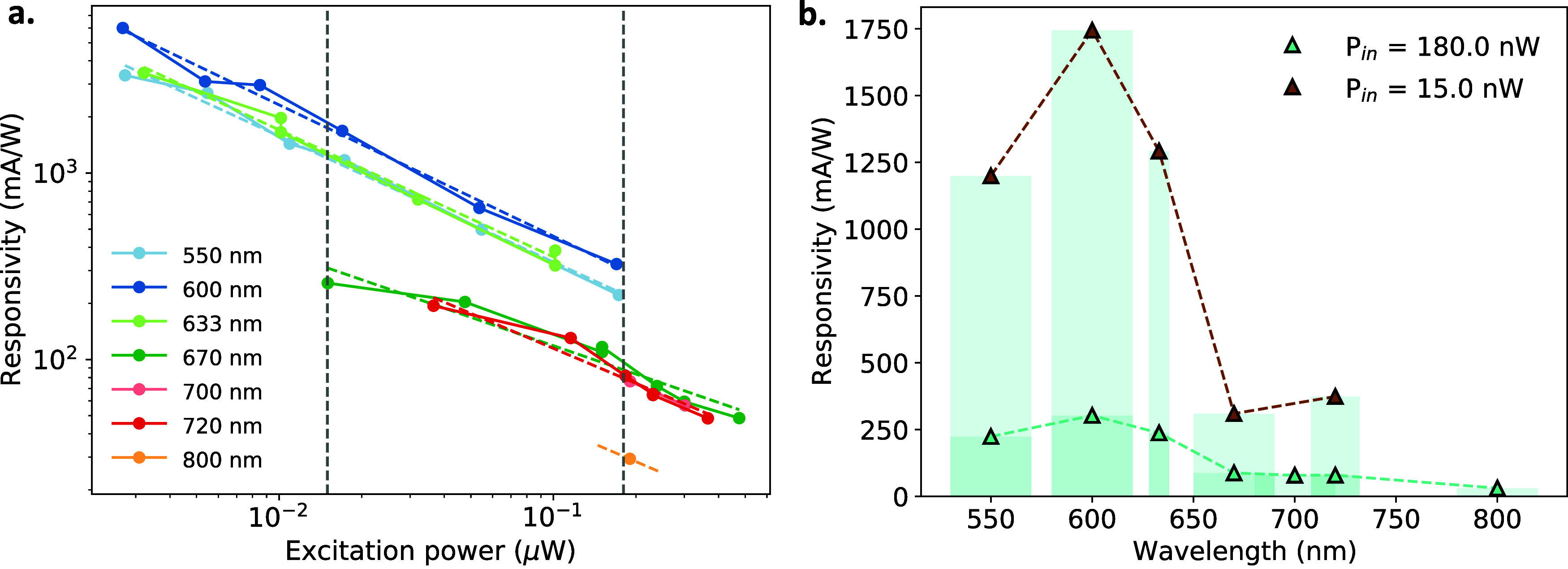
(a) Responsivity as a function of excitation power for different excitation wavelengths, selected by using bandpass filters (550/40, 600/40, 633/10, 670/40, 700/40, 720/24, and 800/40 nm). All curves show a sublinear power dependence with a typical slope of ∼−0.6 in the log–log scale. For 800 nm excitation the responsivity is low and only one point of excitation power is measured. The yellow dashed line in that case is not a fit but a line with a slope of −0.6 serving as a guide to the eye for the expected progression of the responsivity around that point. (b) Responsivity as a function of the excitation wavelength for two representative powers. The horizontal bars represent the full-width-half-maximum (FWHM) of each filter. The responsivity sharply drops beyond the WS_2_ exciton resonance (∼633 nm), consistent with a wavelength-dependent enhancement mechanism due to the WS_2_ optical antenna.

### III.II Mechanism Driving *R*-Enhancement

In order to explore the mechanisms of the 18-fold enhancement observed in our measurements, we perform further theoretical and experimental studies. To investigate the role of WS_2_ in the photoresponse enhancement, we model the layer-resolved absorption of the system. For this purpose, we develop a computational framework which applies the Transfer Matrix Method (TMM) for multilayered structures.^19^ This model allows us to compute the electric field distribution within each layer and subsequently determine the absorbed power density. Figure 4a shows a schematic of the model used, and Figure 4b shows the absorption density as a function of position for two scenaria, for a material stack with and without WS_2_. First observation is that the absorption of the Graphene/MoSe_2_/Graphene system is not significantly altered due to the presence of WS_2_. In fact, it is slightly reduced by 0.98. This means that the observed *R*-enhancement is not a trivial optical effect of increased absorption at the photodetector due to the dielectric modulation of the environment. Second observation is that the WS_2_ layer contributes to the overall absorption of the system by around 27%. This is in stark contrast with the 18-fold *R*-enhancement observed in measurements. By using the same methodology, we extend our study to different wavelengths calculating in that way the absorption spectrum for the two scenaria. The calculated absorption is presented in Figure 4c for the cases with and without WS_2_. The total absorbed power is obtained by integrating the data over the relevant thickness range. Details on the calculation can be found in the Supporting Information Section II.

**Figure 4:**
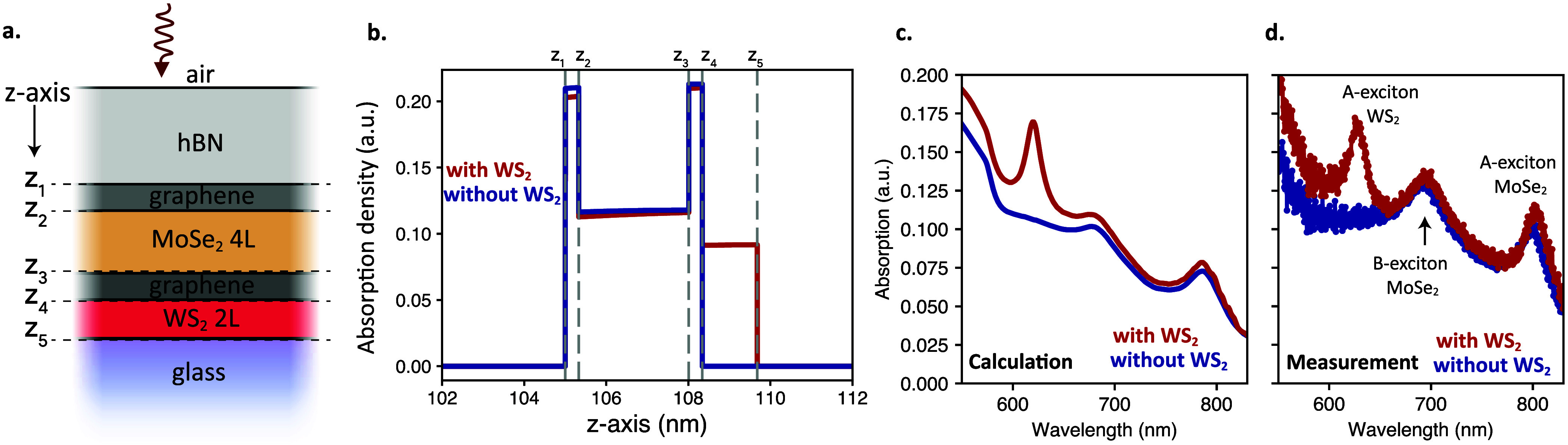
(a) Model of the layered structure considered in the calculations. To perform comparative studies, the layer of WS_2_ is substituted with glass in the cases where no WS_2_ is considered. The dielectric function of the materials used is taken from the literature.^20–22^ (b) Calculated absorption density as a function of distance over the *z*-axis of the device structure. The *z*_1_ to *z*_5_ points refer to the points of the structure presented in panel (a). (c) Calculated absorption spectrum for the whole device structure with and without the WS_2_ layer. (d) Absorption spectrum measured at selected locations to compare Region A (without WS_2_) and Region B (with WS_2_).

To experimentally investigate the absorptive behavior of the proposed structure, we perform optical measurements using a broadband light source and record the spectral transmission (Trans.) with a spectrometer. The reflection (Refl.) is also measured by illumination through the objective. The resulting absorption (*A*) is calculated by using the relation *A* = 1 – Refl. – Trans. which can give an estimation of the absorption in the device. In Figure 4d, we present the absorption data for two illumination positions at Regions A and B (without and with WS_2_). In both regions, the spectra exhibit peaks near the excitonic resonances of MoSe_2_ (A- and B-excitons) and increases at shorter wavelengths. In the region containing WS_2_, we observe an additional peak at the A-exciton resonance of WS_2_.^3^ We find good agreement between calculated and measured curves (Figure 4c,d), strengthening the validity of our calculations. Our calculations reveal that the absorption on the Graphene/MoSe_2_/Graphene photodetector at the illumination wavelength (dashed line in Figure 4d) due to WS_2_ represents only 27% of the total absorption. This indicates that the far-field absorption enhancement is not sufficient to facilitate the observed 18-fold *R*-enhancement. This is a surprising finding whose origin we explore with the following analysis.

Upon illumination near the A-exciton peak of WS_2_, photocarriers are generated in both WS_2_ and MoSe_2_ (Figure 5a). For MoSe_2_, the photon energy is close to the electronic bandgap, making possible the excitation of free carriers. For WS_2_, free-carrier generation is not possible since the electronic bandgap exceeds the photon energy.^3^ Photoexcited WS_2_ excitons cannot be dissociated in the absence of an applied electric field since WS_2_ resides outside the graphene electrodes and there is no potential gradient across the WS_2_ layer.

**Figure 5:**
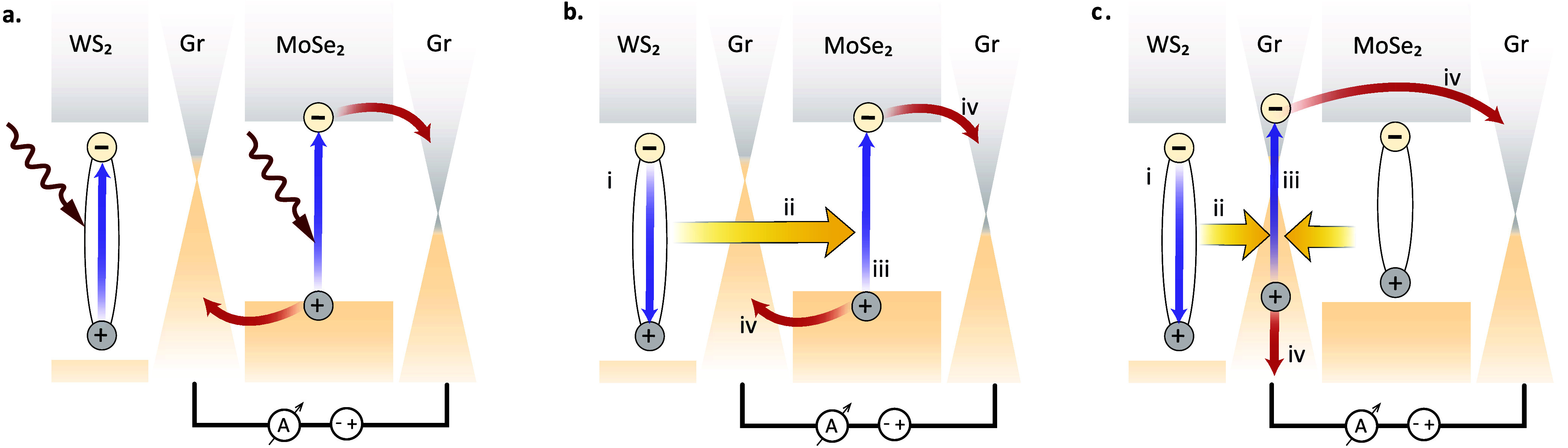
Band diagrams highlighting the charge transport and energy transfer in the device with a WS_2_ top layer. (a) Excitation of photocarriers in MoSe_2_ and WS_2_ by the incidence light, (b) energy transfer from WS_2_ excitons to MoSe_2_. WS_2_ excitons can decay via (i) direct recombination or (ii) energy transfer to MoSe_2_, giving rise to (iii) the generation of electrons and holes in MoSe_2_ and (iv) contributing to the overall photocurrent. (c) Similar process as in (b) but based on energy transfer from WS_2_ to graphene.

A candidate mechanism that does not rely on electronic but only near-field optical coupling is energy transfer. WS_2_ excitons can excite electron–hole pairs in graphene and MoSe_2_ through dipole–dipole (Coulomb) interactions. Such a process depends on the LDOS seen from the WS_2_ excitons. The LDOS in this case is influenced by the presence of graphene and MoSe_2_, especially at energies above the optical bandgap of MoSe_2_. This facilitates the enhanced photoresponse. This process is illustrated in Figure 5b,c (yellow arrows), and is analogous to Förster resonance energy transfer (FRET).^23–25^ Photoresponse on MoS_2_ driven by FRET from cyanine dyes^26^ and the impact of FRET from core–shell quantum dots to graphene or MoS_2_ on absorption properties^27^ are studied in literature. Additionally, FRET has been identified as a mechanism for enhanced photoluminescence in TMD heterostructures^9^ and for photoluminescence quenching in TMDs placed on top of graphene.^28^ While both FRET and charge transfer are known to occur in TMD/graphene heterostructures, we focus our discussion on FRET as the dominant mechanism in our system under near-resonant excitation. This focus is motivated by previous experimental and theoretical studies that conclude energy transfer is often the primary coupling pathway, with sub-5 ps time scales dominating over charge transfer at TMD/graphene interfaces.^28–30^ Although charge transfer may contribute to the overall responsivity observed in our broadband spectral response measurements (Figure 3), we identify FRET as the primary mechanism driving the enhanced photoresponse under near-resonant excitation, where the responsivity is the highest. This interpretation is consistent with previous studies highlighting the dominance of ultrafast energy transfer over charge transfer in TMD/graphene heterostructures.

In our case, the energy of WS_2_ excitons is transferred to MoSe_2_ and graphene (Figure 5b,c, respectively), where it generates electron–hole pairs that are separated by the potential difference between the graphene electrodes. WS_2_, in this case, acts as an optical antenna that concentrates the absorbed optical field in the form of a polarization field (exciton)^31^ and directs it through near-field coupling to excite additional photocarriers in the Graphene/MoSe_2_/Graphene heterostructure, thereby enhancing the photoresponse. In fact, this energy transfer process to graphene can also occur from excited MoSe_2_ excitons as depicted in Figure 5c. However, the excitation of photocarriers from the WS_2_ excitons appears to be more efficient taking into account the observed 18-fold *R*-enhancement and the absence of such enhancement at the measured and calculated absorption spectra (Figure 4c,d).

The significant difference between excitations by photons or through ET from excitons is that while photons are limited to coupling within the light cone, the electromagnetic near field of excitons contains significantly higher in-plane momentum components. This means that, beyond the direct radiative decay channels accessible to photons, the excitonic near field can also couple to indirect, high-momentum absorption channels in graphene (see Figure 6a). In fact, by calculating the angular spectrum of the LDOS of a dipole close to a graphene layer (Figure 6b), we observe that a large portion of the spectrum lies at higher in-plane momenta (outside the light cone), corresponding to nonradiative decay channels. These channels are accessible through the near field of WS_2_ excitons, increasing the probability of energy transfer to graphene. In other words, the WS_2_ excitons quench strongly toward graphene. Similar nonradiative decay is expected for MoSe_2_ excitons as shown in Figure 5c. However, this effect is expected to be less pronounced in the case of 4L-MoSe_2_ due to the increased dielectric screening associated with the thicker flake. Indeed, the LDOS of a dipole near a TMD decreases as the TMD thickness increases, as has been reported both theoretically and experimentally.^27,32,33^ Conversely, the opposite trend has been reported for graphene,^27^ making WS_2_ excitons more likely to decay toward graphene than MoSe_2_ excitons, and thus contributing more strongly to the photocurrent. This favorable decay of 2L-WS_2_ excitons toward graphene due to the decreased dielectric screening, compared to 4L-MoSe_2_ excitons, is depicted in Figure 6c and contributes to the observed *R*-enhancement.

**Figure 6:**
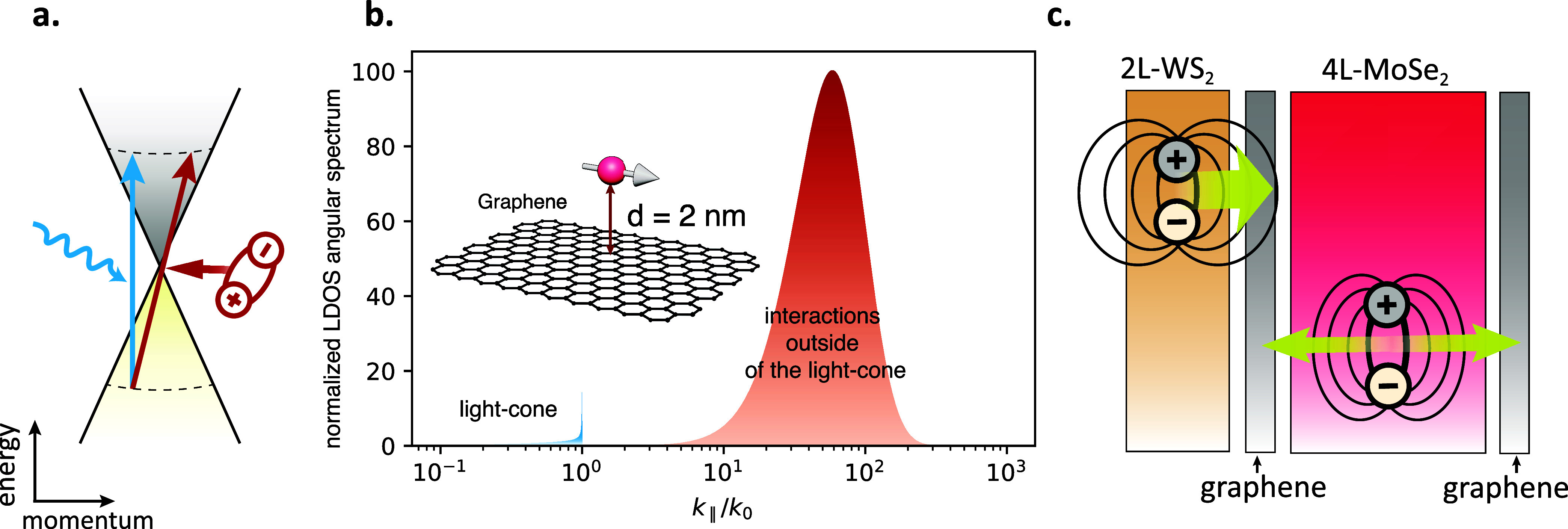
(a) Illustration of the direct transitions on the graphene band structure at the *K*-point mediated by photon absorption and the indirect transition mediated by exciton absorption. (b) Calculation of the angular spectrum of the local density of optical states (LDOS) of a point electric dipole positioned 2 nm away from a graphene layer, highlighting the dominance of high-momentum (nonradiative) components outside of the light cone. The calculation method is outlined in Supporting Information Section II. The dielectric function of graphene is taken from the literature.^20,22^ (c) Illustration of 2L-WS_2_ and 4L-MoSe_2_ exciton quenching toward graphene, demonstrating the stronger energy transfer efficiency of WS_2_ excitons due to reduced dielectric screening and exciton confinement.

Although the previous discussion appears to suggest the use of thin flakes of TMDs in photodetectors for enhanced energy transfer toward the electrodes, the use of thicker flakes can increase the photon absorption, which can also increase photodetection efficiency, revealing a trade-off between the two. Moreover, the use of thicker flakes as tunnel barriers (4L-MoSe_2_) is also important for keeping the responsivity high by reducing the leakage current. For example, using a 2L-TMD as the tunnel barrier, to enhance energy transfer to graphene the photodetection channel, is not a good design choice, because then the leakage current will substantially reduce the responsivity. In fact using a design where the thin TMD (WS_2_) can sit outside of the junction to contribute to the ET processes and a thick TMD (4L-MoSe_2_) as in our photodetector appears to be a good strategy to optimize lower leakage current through the thick MoSe_2_ layer and still profit from the reduced dielectric screening of 2L-WS_2_ excitons that can quench efficiently toward graphene and enhance the photocurrent.

Concerning the drop of R with increasing power presented in Figure 2d, we note that the energy transfer efficiency depends on the lifetime of WS_2_ excitons and any competing processes. One of them is exciton–exciton annihilation (EEA) known as well as Auger recombination which appears at high pump powers^34^ and is likely the mechanism responsible for the reduction of responsivity shown in Figure 2d. Additionally, the saturation of MoSe_2_ and graphene could also reduce energy transfer efficiency and slow down the interfacial transfer^12^ from MoSe_2_ to graphene. In this case, the photocurrent is probably originating from the free carriers generated in MoSe_2_. At low pump powers, EEA is negligible and the WS_2_ exciton energy transfer does not compete with EEA, contributing that way to the photocurrent of the Graphene/MoSe_2_/Graphene photodetector.

## IV Conclusions

In summary, the enhanced photoresponse enabled by a WS_2_ antenna in the Graphene/MoSe_2_/Graphene photodetector is investigated through spatially resolved scans and responsivity measurements at different *V*_b_ and *P*_in_ values in selected locations. Our measurements show up to 18 times responsivity enhancement at optimal *V*_b_ and *P*_in_. In addition, spectrally resolved responsivity measurements highlight the role of WS_2_ in the enhancement as increased responsivity is observed at excitation energies close to the WS_2_ exciton energy. Our experimental and theoretical analysis allows us to identify energy transfer from WS_2_ excitons to graphene and MoSe_2_ as the driving mechanism of this enhancement. More specifically, the enhancement is attributed to the efficient nonradiative decay of WS_2_ excitons toward the graphene and MoSe_2_ layers. The efficiency of the process relies on the reduced thickness of WS_2_ and the ability of excitons to excite modes outside of the light cone making possible their enhanced absorption from graphene and MoSe_2_.

We recognize the possibility for further studies of the energy transfer rate to further optimize *R*-enhancement. Replacing the bilayer WS_2_ with a monolayer could increase the energy transfer rate at the expense of optical absorption and the process can be further controlled and studied by introducing a spacer layer (such as few-layer hBN) between the graphene and WS_2_. Such approach has been reported to provide a convenient way to study energy transfer processes.^7,9^ We also note that our devices operate in the photoconductive regime, and the dark current can be reduced by increasing the thickness of the junction barrier at the expense of increasing the photocarrier transit time. Further studies with varying MoSe_2_ thicknesses could help in pinpointing the optimal conditions for FRET and achieving enhanced responsivity.

Our work motivates further investigations on the fine balance between the thickness of the antenna-TMD (WS_2_) and the barrier-TMD (MoSe_2_) for optimized photoresponse. While our study primarily serves as a proof of principle, it demonstrates performance comparable to state-of-the-art research in the literature and even to commercial photodiodes. A detailed comparison is provided in Supporting Information Section III.

Finally, we want to highlight that the antenna-like effect of the WS_2_ layer enhances the photoresponse without requiring additional lithographic patterning or integration with quantum dots, nanoparticles, or cavity structures. The ability to control the responsivity with a TMD optical antenna defines a new paradigm for photodetector design based on 2D-heterostructures.

## Supplementary Material



## Notes

Photocurrent maps under applied bias. Photonic calculations of absorbed optical power. Calculation of the optical density of states. Comparison of responsivity with existing TMD-based photodetectors (PDF)

## ∥

Photonic Systems Laboratory, EPFL, 1015 Lausanne, Switzerland

## ⊥

Université de Strasbourg, CNRS, Institut de Physique et Chimie des Matériaux de Strasbourg, UMR 7504, F-67000 Strasbourg, France.

## #

Center for Photonics Sciences, University of Eastern Finland, FI-80101 Joensuu, Finland.
