# Supplementary material for: Van der Waals Photodetector with an Integrated WS2 Light-Harvesting Antenna
Source: ACS Photonics. 2025 Sep 17;12(10):5390–8. doi: 10.1021/acsphotonics.5c00801 (PMC12532370; doi:10.1021/acsphotonics.5c00801)
Supplement: Supplementary file 1 [file ph5c00801_si_001.pdf]

Supporting Information
Van der Waals photodetector with an integrated WS_2 light-harvesting antenna

Yesim Koyaz[1, 2, †], Sotirios Papadopoulos[1,5, †,*], Antti J. Moilanen[1,6], Jonas D. Ziegler[1], Takashi Taniguchi[3], Kenji Watanabe[4], Lujun Wang[1], and Lukas Novotny[1, **]

[1]Photonics Laboratory, ETH Zurich, 8093 Zurich, Switzerland
[2]Current Address: Photonic Systems Laboratory, EPFL, 1015 Lausanne, Switzerland
[3]Research Center for Materials Nanoarchitectonics, National Institute for Materials Science, 1-1 Namiki, Tsukuba 305-0044, Japan
[4]Research Center for Electronic and Optical Materials, National Institute for Materials Science, 1-1 Namiki, Tsukuba 305-0044, Japan
[5] Current address: Université de Strasbourg, CNRS, Institut de Physique et Chimie des Matériaux de Strasbourg, UMR 7504, F-67000, Strasbourg, France
[6] Current address: Center for Photonics Sciences, University of Eastern Finland, FI-80101 Joensuu, Finland and
[*]sotirios.papadopoulos@ipcms.unistra.fr, [**]lukas.novotny@ethz.ch, [†]These authors contributed equally.

I. Photocurrent Maps Under Applied Bias

Following the same methodology described in the main text, we present photocurrent (I_{ph}) maps for different bias voltages (V_b) while raster scanning the laser spot (Fig. S1). For consistency, the same the illumination power (P_{in}) and scan area as in Fig. 2 is used. Under applied bias, we observe that I_{ph} increases with positive V_b and decreases with negative V_b, consistent with the trends discussed in the main text. At $V_b = -0.08$ V, the photocurrent map shows a clear signal in Region-A, while Region-C to F exhibit near-zero response. At $V_b = +0.08$ V, Region-A also shows suppressed photocurrent, which we attribute to compensation of the initial doping in the graphene electrodes at zero bias (further detailed in the main text). At higher biases ($V_b = \pm 0.15$ V), Region-A again shows a prominent photocurrent while Region-C to F show increased noise contributions. Despite the limitations introduced by elevated electrical noise at higher V_b, Region-A (Graphene/$MoSe_2$/Graphene) and Region-B (Graphene/$MoSe_2$/Graphene/WS_2) are distinguishable the primary active regions at various V_b where I_{ph} is particularly prominent on Region B, indicating R-enhancement due to WS_2 bilayer.

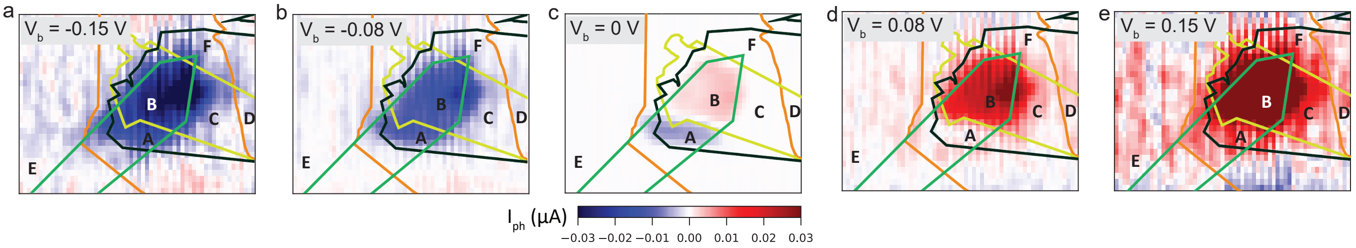

FIG. S1. Photocurrent maps for bias voltage (a) $V_b = -0.15$ V, (b) $V_b = -0.08$ V, (c) $V_b = 0$ V, (d) $V_b = 0.08$ V and (e) $V_b = 0.15$ V, recorded by raster scanning the laser spot. All plotted using the same color scale. Region-A and Region-B are Graphene/$MoSe_2$/Graphene and WS2/Graphene/$MoSe_2$/Graphene heterostructures, respectively. Region-C, D and F corresponds to WS_2/Graphene/$MoSe_2$, WS_2/Graphene and $MoSe_2$/Graphene, respectively. Region-E is labeled solely as a control region for reference.

In Fig. 6, we perform measurements on a second sample which is composed of WS_2/Graphene/$MoSe_2$/Graphene. To evaluate the quality of this sample, we use optical microscopy images and position dependent photocurrent maps. It is fabricated by using 2-layer WS_2 and 3-layer $MoSe_2$ flakes following the same procedure described in the main text. The WS_2 layer fully covers the active area of the device. As an initial characterization, we illuminate the device with the same HeNe laser beam and scan the beam's position across the region marked by the white rectangle in Fig. S2b. The resulting photovoltaic photoresponse is shown in Fig. S2c, and the corresponding photocurrent data at bias voltages $V_b = 0.3$ V and $V_b = -0.3$ V are presented in Fig. S2d and Fig. S2e, respectively. In terms of photocurrent magnitude, this sample exhibits a comparable response (even slightly higher) to the first sample at the respective bias voltages, as evident from a comparison between Fig. 2a and Fig. S2d.

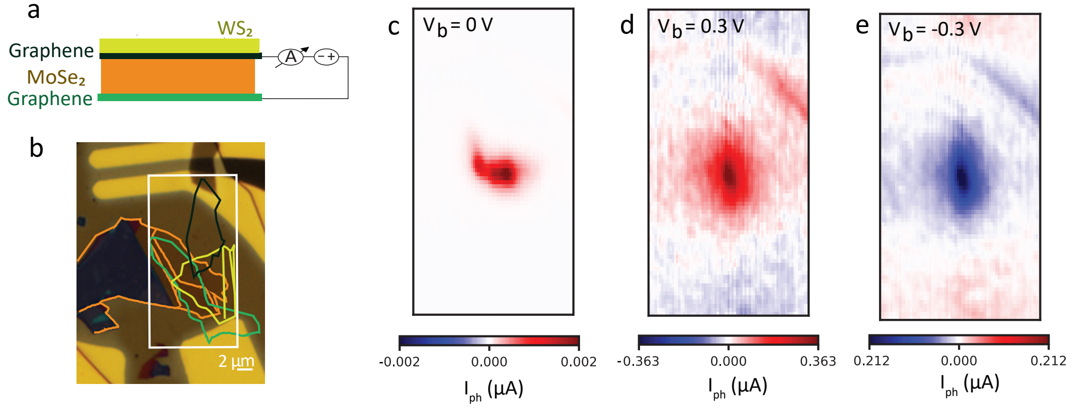

FIG. S2. Photoresponse of second sample (a) Its cross-sectional schematic containing Graphene/$MoSe_2$/Graphene vertical junction with WS_2 top layer. (b) Optical image of the characterized photodetector where the outlines of the exfoliated 2D-flakes and the measurements' scan area (white rectangle) are labeled. (c-e) Photocurrent maps for bias voltage (c) V_b = 0 V, (d) V_b = 0.3 V, (e) V_b = −0.3 V, recorded by raster scanning the laser spot.

II. Photonic calculations of absorbed optical power

Here we evaluate whether the observed enhancement in responsivity is due to trivial optical effects, such as an increase in absorbed power caused by modifications in the dielectric environment imposed by the presence of a WS_2 bilayer. The absorbed power in a specific layer of a multilayered structure can be calculated using the Transfer Matrix Method (TMM), which is widely used for solving Maxwell's equations in stratified media. To facilitate such calculations, we have developed a Python package, available in GitHub [1].

This package provides an implementation of TMM, allowing for the computation of the electric field distribution $E(z)$ inside the layers. The absorbed power density $Q(z)$ per unit volume in a lossy dielectric medium with complex permittivity $\varepsilon = \varepsilon' + i\varepsilon''$ is given by

$$Q(z) = \frac{1}{2}\omega\varepsilon_0 \mathrm{Im}(\varepsilon)|E(z)|^2 \tag{1}$$

where $\omega = 2\pi f$ is the angular frequency, ε_0 is the permittivity of free space, $\mathrm{Im}(\varepsilon)$ is the imaginary part of the permittivity, which accounts for absorption, and $E(z)$ is the local electric field at position z.

The total absorbed power P_{abs} in a layer of thickness d is obtained by integrating the absorbed power density over the volume of the layer,

$$P_{\mathrm{abs}} = \int_{\mathrm{layer}} Q(z)\,dV. \tag{2}$$

For a plane wave propagating along the z-direction, this simplifies to

$$P_{\mathrm{abs}} = \frac{1}{2}\omega\varepsilon_0 \mathrm{Im}(\varepsilon)\int_0^d |E(z)|^2\,dz. \tag{3}$$

We consider the systems shown in Fig. S3, where one includes a WS_2 bilayer and the other does not. The electric field inside each layer is determined using the Transfer Matrix Method (TMM), as implemented in our Python package. The field can be expressed as a sum of forward- and backward-propagating waves,

$$E(z) = Ae^{ik_z z} + Be^{-ik_z z}, \tag{4}$$

where A and B are the field coefficients obtained from the boundary conditions using TMM, and k_z is the propagation constant inside the layer. By substituting $|E(z)|^2$ into the integral for P_{abs}, we obtain the total absorbed power in the layers of interest. Specifically, we focus on the photodetector region, which consists of the graphene/$MoSe_2$-4L/graphene stack in the range $z_1 < z < z_4$, where the absorbed power is given by

$$P_{\mathrm{abs}} = \frac{1}{2}\omega\varepsilon_0 \mathrm{Im}(\varepsilon)\int_{z_1}^{z_4} |E(z)|^2 dz. \tag{5}$$

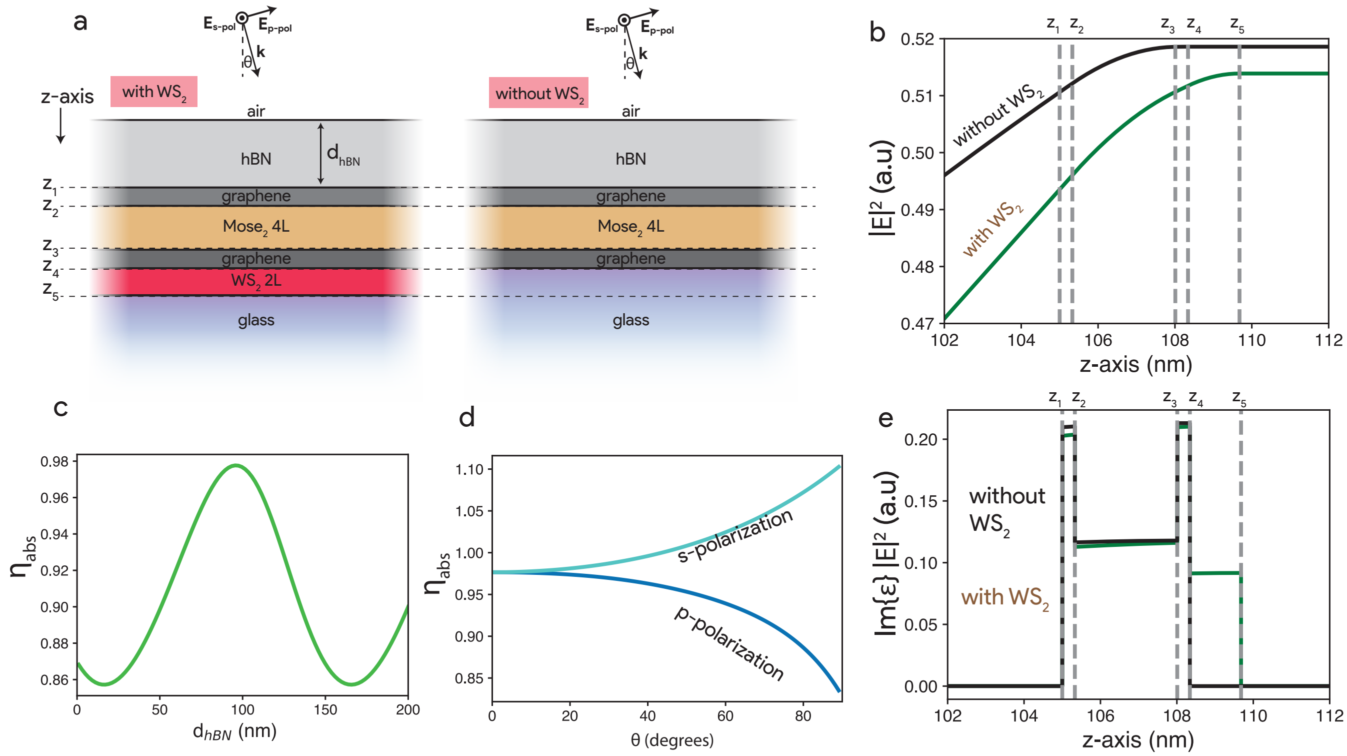

FIG. S3. (a) Schematic representation of the system with and without the WS_2 bilayer. (b) Electric field intensity distribution $|E(z)|^2$ for the two cases at normal incidence ($\theta = 0$). (c) Absorption enhancement ratio η_{abs} as a function of hBN thickness. (d) η_{abs} as a function of the incidence angle θ for s- and p-polarized light.(e) Electric field intensity distribution multiplied by the imaginary part of the dielectric function of the structure representing the absorption at the different layers of the device.

By performing this calculation for both systems, we can compute the absorption enhancement ratio, which quantifies the increase in absorbed power due to the presence of the WS_2 bilayer,

$$\eta_{\text{abs}} = \frac{P_{\text{abs}}^{\text{WS}_2}}{P_{\text{abs}}^{\text{no WS}_2}} \tag{6}$$

where $P_{\text{abs}}^{\text{WS}_2}$ is the absorbed power in the presence of the WS_2 layer, and $P_{\text{abs}}^{\text{no WS}_2}$ is the absorbed power in the absence of WS_2. The squared field distribution $|E(z)|^2$ for both cases at normal incidence ($\theta = 0$) and for p-polarized light at a wavelength of 633 nm is shown in Fig. S3b. The results indicate that the presence of WS_2 slightly reduces the field intensity in the photodetector region, leading to an absorption enhancement ratio of $\eta_{\text{abs}} = 0.98$.

The thickness of the top hBN layer is estimated to be approximately 100 nm based on optical contrast. However, given the potential uncertainty in this estimation, we calculate η_{abs} for a range of hBN thicknesses, as shown in Fig. S3c. The variation of η_{abs} remains minimal, confirming that absorption is weakly dependent on hBN thickness.

We further analyze η_{abs} for different angles of incidence and polarizations. Fig. S3d and S3e present the calculated angle-dependent η_{abs} for s- and p-polarized light in the range $0^\circ < \theta < 90^\circ$. The results show that η_{abs} remains close to unity and exceeds 1 only for high-angle s-polarized light, a regime that is not accessible in our experimental setup (NA = 0.9).

Overall, our calculations indicate that the observed responsivity enhancement cannot be attributed to trivial optical effects related to absorption enhancement due to dielectric-environment modulation. The WS_2 layer does not significantly modify the absorbed power within the photodetector, suggesting that other mechanisms must be responsible for the enhanced responsivity.

As a further step in our theoretical study of absorption, we calculate the product $Im\{\varepsilon\}|E|^2$, which is related to the absorption at every point of the structure, as highlighted by Eq. 5. The result is presented in Fig. S3 for the cases with and without WS_2 layer. This calculation reveals that the absorption associated with WS_2 represents only 27% of the total absorption. This is an interesting result suggesting that the photocurrent enhancement is not related directly to the absorption of WS_2 and shows that the enhancement relates on efficient energy transfer between the WS_2 and the photodetector.

III. Calculation of the optical density of states

The dissipated power P of a point electric dipole is calculated by [2]

$$P = \frac{1}{2}\omega \, \mathbf{p} \, Im\{\mathbf{E}(\mathbf{r_0})\} \tag{7}$$

where ω is the angular frequency of the dipole's emission, **p** is the dipole moment and **E** is the electric field at the dipoles origin r_0. The dipole is assumed to be situated at the center of the tunneling layer. The electric field $\mathbf{E}(\mathbf{r}_0)$ is calculated by solving the electromagnetic wave equation for a multi-layer structure. The optical density of states is then calculated through its relation to P [2]

$$\frac{\rho_{\text{opt}}}{\rho_0} = \frac{P}{P_0} \tag{8}$$

with P_0 being the dissipated power for a point dipole in vacuum.

IV. Comparison of Responsivity with Existing TMD-based Photodetectors

Here we provide a detailed comparison with similar Graphene-TMD-Graphene heterostructures reported in the literature, focusing on cases that closely resemble our device (without the WS_2 antenna). We specifically compare responsivity values to highlight how our approach stands in relation to other van der Waals heterostructures:

1. Graphene-MoS_2(2L)-WSe_2(2L)-Graphene Heterostructure [3]

Responsivity: ~150 mA/W at a voltage per thickness of ~2 mV/nm
Comparison: Our device achieves a peak responsivity of $<$ 35 mA/W at $V_b = 0.3$ V, corresponding to 0.1 V/nm (assuming a $MoSe_2$ thickness of ~2.8 nm).

2. Graphene-WSe_2-Graphene Heterostructure [4]

External Quantum Efficiency (EQE): ~7.3% for a 2.2-nm-thick device with three layers of WSe_2
Comparison: Our device achieves a comparable EQE of ~7% for a $MoSe_2$ thickness of 2.8 nm (calculated using $EQE = \frac{Rhc}{e\lambda}$ [5]). This value is obtained at $V_b = 0.3$ V.

3. Graphene-$MoTe_2$-Graphene Heterostructure (~10 nm $MoTe_2$ thickness) [6]

Responsivity: 3.4 mA/W at 1064 nm under a back-gate voltage of 30 V
Comparison: Due to differences in the gating/biasing scheme, direct comparison is challenging. However, our device achieves a responsivity ($<$ 35 mA/W) within the same order of magnitude, indicating that our approach remains competitive.

Overall, the Graphene/$MoSe_2$/Graphene photodetector demonstrates performance comparable to the previously reported heterostructures in the literature. The WS_2-enhanced photodetector achieves up to 0.6 A/W at $V_b = 0.3$ V, demonstrating the viability of LDOS engineering and energy transfer mechanisms to enhance photodetection. While we recognize the photoconductive nature of our response, further improvements are possible by increasing the applied bias or incorporating a gating scheme. For context, we also compare our responsivity to commercial silicon photodiodes, such as the Thorlabs FDS010 [7], which typically exhibit responsivities in the range of 0.4–0.7 A/W at visible wavelengths. While direct comparison with commercial photodiodes on different parameters like noise, dark current or photogain is out-of-scope for this work, our results remain promising and indicate potential for further enhancement through improved device engineering.

Additionally, we also provide a comparison between our approach and some other photoresponse enhancement techniques including QDs and plasmonic structures integrated into 2D-photodetectors in the following table. We acknowledge that our comparison does not capture all existing studies but aims to provide a general perspective.

Reference	Heterostructure Materials	Enhancement vs. baseline
[8]	Au nanoparticle core with CVD-grown multi-layer MoS_2 shell	~10× higher R than planar MoS_2 transistor

[9]	Ag nanoparticle-doped n-WS_2 on p-Si heterojunction	Significantly higher than a commercial Si photodiode (8.0 A/W vs ∼0.5 A/W, ∼16×)
[10]	WS_2 nanodisk/graphene stack on AgNP metafilm	∼5× higher R with Ag NPs
[11]	Monolayer MoS_2 with Ag shell-isolated nanospheres an top of Au film	880% photocurrent increase compared to monolayer MoS_2 alone (SiO_2 substrate)
[12]	Au nanoparticle – MoS_2 – Au nanoparticle stack	3× higher than that of the only one-layered Au NP
[13]	TDBC J-aggregate (donor) + monolayer MoS_2 (acceptor) in FET cavity (h-BN spacer)	Up to 14× improvement on photoresponsivity
[14]	p-Si/WS_2 quantum dots Schottky photodiode, with vs. without embedded Au nanoparticles	∼10× enhancement in responsivity with Au NPs
[15]	WS_2 nanodiscs on graphene (vs. continuous WS_2 film on graphene)	7× responsivity enhancement
[16]	WS_2/MoS_2 vertical van der Waals heterostructure, with vs. without Au nanoparticles (SPR enhancement)	∼25× responsivity enhancement in IR
**This work**	**WS_2 antenna on Graphene/$MoSe_2$/Graphene heterojunction**	**Up to 18× enhancement**

Overall, our results demonstrate that responsivity enhancement through WS_2 excitonic energy transfer is a promising approach that offers improved performance compared to similar TMD-based photodetectors and achieves enhancement levels comparable to other state-of-art responsivity enhancement techniques.

REFERENCES

[1] S. Papadopoulos, "photonic_sim v1.0 : A python module for photonic simulations." 10.5281/zenodo.15021919, Accessed 26 Aug. 2025.

[2] L. Novotny and B. Hecht, "Principles of nano-optics," *Principles of Nano-Optics*, vol. 9781107005464, pp. 1–564, 1 2009.

[3] C.-H. Lee, G.-H. Lee, A. M. Van Der Zande, W. Chen, Y. Li, M. Han, X. Cui, G. Arefe, C. Nuckolls, T. F. Heinz, *et al.*, "Atomically thin p–n junctions with van der waals heterointerfaces," *Nature nanotechnology*, vol. 9, no. 9, pp. 676–681, 2014.

[4] M. Massicotte, P. Schmidt, F. Vialla, K. G. Schädler, A. Reserbat-Plantey, K. Watanabe, T. Taniguchi, K. J. Tielrooij, and F. H. Koppens, "Picosecond photoresponse in van der Waals heterostructures," *Nature nanotechnology*, vol. 11, pp. 42–46, 1 2016.

[5] K. Kumar and D. Kaur, "A review on recent advancements in the growth of mos_2 based flexible photodetectors," *Solar Energy Materials and Solar Cells*, vol. 268, p. 112736, 2024.

[6] K. Zhang, X. Fang, Y. Wang, Y. Wan, Q. Song, W. Zhai, Y. Li, G. Ran, Y. Ye, and L. Dai, "Ultrasensitive Near-Infrared Photodetectors Based on a Graphene-$MoTe_2$-Graphene Vertical van der Waals Heterostructure," *ACS Applied Materials and Interfaces*, vol. 9, pp. 5392–5398, 2 2017.

[7] Thorlabs, "Thorlabs fds010 - si photodiode, 1 ns rise time, 200 - 1100 nm, Ø1 mm active area." https://www.thorlabs.com/thorproduct.cfm?partnumber=FDS010, 2025. Accessed: 14-Mar-2025.

[8] Y. Li, J. G. DiStefano, A. A. Murthy, J. D. Cain, E. D. Hanson, Q. Li, F. C. Castro, X. Chen, and V. P. Dravid, "Superior plasmonic photodetectors based on au@ mos_2 core–shell heterostructures," *ACS nano*, vol. 11, no. 10, pp. 10321–10329, 2017.

[9] R. Chowdhury, T. Sinha, A. Katiyar, and S. Ray, "Synergistic effect of polymer encapsulated silver nanoparticle doped ws_2 sheets for plasmon enhanced 2d/3d heterojunction photodetectors," *Nanoscale*, vol. 9, no. 40, pp. 15591–15597, 2017.

[10] M. Alamri, B. Liu, S. M. Sadeghi, D. Ewing, A. Wilson, J. L. Doolin, C. L. Berrie, and J. Wu, "Graphene/ws_2 nanodisk van der waals heterostructures on plasmonic ag nanoparticle-embedded silica metafilms for high-performance photodetectors," *ACS Applied Nano Materials*, vol. 3, no. 8, pp. 7858–7868, 2020.

[11] Z.-Q. Wu, J.-L. Yang, N. K. Manjunath, Y.-J. Zhang, S.-R. Feng, Y.-H. Lu, J.-H. Wu, W.-W. Zhao, C.-Y. Qiu, J.-F. Li, *et al.*, "Gap-mode surface-plasmon-enhanced photoluminescence and photoresponse of mos_2," *Advanced Materials*, vol. 30, no. 27, p. 1706527, 2018.

[12] G. Li, Y. Song, S. Feng, L. Feng, Z. Liu, B. Leng, Z. Fu, J. Li, X. Jiang, B. Liu, *et al.*, "Improved optoelectronic performance of mos_2 photodetector via localized surface plasmon resonance coupling of double-layered au nanoparticles with sandwich structure," *ACS Applied Electronic Materials*, vol. 4, no. 4, pp. 1626–1632, 2022.

[13] J. Dutta, N. Yadav, P. Bhatt, K. Kaur, D. E. Gómez, and J. George, "Enhanced energy transfer in cavity qed based phototransistors," *The Journal of Physical Chemistry Letters*, vol. 15, no. 32, pp. 8211–8217, 2024.

[14] A. Bora, L. P. Mawlong, and P. Giri, "Highly suppressed dark current and fast photoresponse from au nanoparticle-embedded, si/au/ws2 quantum-dot-based, self-biased schottky photodetectors," *ACS Applied Electronic Materials*, vol. 3, no. 11, pp. 4891–4904, 2021.

[15] M. Alamri, M. Gong, B. Cook, R. Goul, and J. Z. Wu, "Plasmonic ws_2 nanodiscs/graphene van der waals heterostructure photodetectors," *ACS applied materials & interfaces*, vol. 11, no. 36, pp. 33390–33398, 2019.

[16] G. Wang, L. Li, W. Fan, R. Wang, S. Zhou, J.-T. Lü, L. Gan, and T. Zhai, "Interlayer coupling induced infrared response in ws_2/mos_2 heterostructures enhanced by surface plasmon resonance," *Advanced Functional Materials*, vol. 28, no. 22, p. 1800339, 2018.
